# Cross-talks among *GBA* mutations, glucocerebrosidase, and α-synuclein in *GBA*-associated Parkinson’s disease and their targeted therapeutic approaches: a comprehensive review

**DOI:** 10.1186/s40035-020-00226-x

**Published:** 2021-01-15

**Authors:** Tapan Behl, Gagandeep Kaur, Ovidiu Fratila, Camelia Buhas, Claudia Teodora Judea-Pusta, Nicoleta Negrut, Cristiana Bustea, Simona Bungau

**Affiliations:** 1 Chitkara College of Pharmacy, Chitkara University, Rajpura, Punjab India; 2 Department of Medical Disciplines, Faculty of Medicine and Pharmacy, University of Oradea, Oradea, Romania; 3 Department of Morphological Disciplines, Faculty of Medicine and Pharmacy, University of Oradea, Oradea, Bihor County Romania; 4 Department of Psycho-Neuroscience and Recovery, Faculty of Medicine and Pharmacy, University of Oradea, Oradea, Romania; 5 Department of Preclinical Disciplines, Faculty of Medicine and Pharmacy, University of Oradea, Oradea, Romania; 6 Department of Pharmacy, Faculty of Medicine and Pharmacy, University of Oradea, Oradea, Romania

**Keywords:** Parkinson’s disease, Glycosylceramidase, Glucocerebrosidase, Gaucher’s disease, Mutations, α-Synuclein

## Abstract

Current therapies for Parkinson’s disease (PD) are palliative, of which the levodopa/carbidopa therapy remains the primary choice but is unable to modulate the progression of neurodegeneration. Due to the complication of such a multifactorial disorder and significant limitations of the therapy, numerous genetic approaches have been proved effective in finding out genes and mechanisms implicated in this disease. Following the observation of a higher frequency of PD in Gaucher’s disease (GD), a lysosomal storage condition, mutations of glycosylceramidase beta (*GBA*) encoding glucocerebrosidase (GCase) have been shown to be involved and have been explored in the context of PD. *GBA* mutations are the most common genetic risk factor of PD. Various studies have revealed the relationships between PD and *GBA* gene mutations, facilitating a better understanding of this disorder. Various hypotheses delineate that the pathological mutations of *GBA* minimize the enzymatic activity of GCase, which affects the proliferation and clearance of α-synuclein; this affects the lysosomal homeostasis, exacerbating the endoplasmic reticulum stress or encouraging the mitochondrial dysfunction. Identification of the pathological mechanisms underlying the *GBA*-associated parkinsonism (GBA + PD) advances our understanding of PD. This review based on current literature aims to elucidate various genetic and clinical characteristics correlated with *GBA* mutations and to identify the numerous pathological processes underlying GBA + PD. We also delineate the therapeutic strategies to interfere with the mutant GCase function for further improvement of the related α-synuclein–GCase crosstalks. Moreover, the various therapeutic approaches such as gene therapy, chaperone proteins, and histone deacetylase inhibitors for the treatment of GBA + PD are discussed.

## Background

Parkinson’s disease (PD) is the second most common neurodegenerative disease that affects 2%–3% of the elderly (above 65 years) around the world, which is characterized by progressive dopaminergic neuronal degeneration in the substantia nigra and α-synuclein aggregation. PD is clinically defined as the combination of restful tremor, rigidity, bradykinesia, and postural instability [1–4]. Research advances in human genetics have significantly improved the understanding of PD. As PD is a multifactorial disorder and current therapies have significant limitations, numerous genetic approaches have been proven effective in sequencing the whole genome, which has led to the recognition of unpredictable genes and mechanisms implicated in this disease. Nevertheless, the most important genetic risk element in the case of PD in trials of patients with an uncommon lysosomal storage condition is the “Gaucher’s disease (GD)” [5]. The glycosylceramidase beta (*GBA*) gene located on chromosome 1 (1q21) encodes glucocerebrosidase (GCase), a lysosomal enzyme engaged in the glucosylceramide metabolism. Mutations in the gene have been widely correlated with GD, which is an inherited, systemic disorder with a significant level of central nervous system involvement [6–9]. Amazingly, research in the past 14 years has suggested that mutations within this gene are linked with elevated incidence of PD as well as the PD incidence in GD patients and asymptomatic carriers [2]. GCase is involved in the endolysosomal path, which appears to be essential in PD pathogenesis, in which a link among PD, *GBA* mutations, and GCase function has been discovered through clinical observations and the genes engaged in this process are responsible for several specific monogenic familial variants of PD [10, 11]. *GBA* gene mutations are much more persistent in most PD populations than other genes involved such as α-synuclein *SNCA*, *PARK2*, and *LRKK2* [12, 13]. Mutations of *GBA* are the most significant risk factor for PD and the variants of *GBA* can raise the PD risk by up to 10 folds [7, 14]. Nowadays, over 300 mutations have been identified in *GBA* [14–16]. It has been proposed that not only a persistent lack of action of GCase enzyme, but also the probable toxic retain-of-function of mutant GCase, may lead to lysosome dysfunction and endoplasmic reticulum (ER) stress that facilitate the disease pathogenesis [17]. In addition, mutant GCase may not fold appropriately and will thus concentrate in various dopaminergic neuronal cells, triggering cellular stress, which can cause further damage to the cells; however, impeded activity of GCase tends to cause the aggregation of α-synuclein [18]. In addition to the contribution of *GBA* in PD development, researchers have highlighted different clinical characteristics in PD patients with *GBA* mutations as compared to the idiopathic PD [19].

In this review, we outline how GCase was recognized as the risk element for PD, the involvement of numerous molecular pathways in GBA + PD, and the identification of clinically relevant and new fields of PD research and therapies arising from this finding. Also, we summarize the genetic alterations and factors linked with the gene mutations in GD and PD and the associated clinical features. GCase deficiency leads to α-syn accumulation, and α-syn in turn can inhibit GCase activity, thereby enhancing α-syn aggregation. It will help to navigate a key role of *GBA* mutations in the pathogenesis. Hereby, we review the current knowledge on the GCase-α-synuclein pathway as a therapeutic target for the sporadic PD type. The most important literature was searched in order to explore the most recent and relevant published papers in the field.

## Main text

### An overview: *GBA* gene and GCase

The *GBA* gene encoding the lysosomal enzyme GCase comprises 10 introns and 11 exons and is located on chromosome 1q21, which is a gene-rich site composed of 2 pseudogenes (*GBAP* and *MTX1*) and 9 genes within a long sequence of about 100 kb. The highly untranslated pseudogene *GBAP* is homologous to *GBA*, which share a 96% exonic sequence identity in the coding region, complicating the detection of *GBA* mutations. The second convergently transcribed pseudogene *MTX1* (Metaxin-1) encodes a protein found in the mitochondrial external (outer) membrane and is located downstream to the *GBAP* sequence [5, 12, 13, 20–22]. GCase (*D*-glucosyl-*N*-acylspingosine glucohydrolase) is a 497-amino-acid (AA) active protein with 5 glycosylation regions [17, 23–27]. The protein is synthesized in the ER, transported by the lysosomal integral membrane protein-2 (LIMP2), which is a GCase transporter encoded by *SCARB2* gene, and becomes active upon reaching the acidic lumen of the lysosome by interacting with its activator protein saposin C. In the lysosomal compartment, the enzyme hydrolyses glucose moieties from glucosylceramide and glucosylsphingosine [21]. GCase consists of 3 domains: domain-1, an antiparallel β-sheet; domain-2, an immunoglobulin-like domain comprised of two closely associated β sheets; and domain-3, a (β/α)8 triosephosphate isomerase (TIM) barrel [17, 23].

GCase goes through several structural alterations during the transportation to the lysosome. It has 2 functional ATG-initiation sites, transcribed as a 516- or 536-AA protein, which is further processed into the 497-AA functional enzyme [23, 28]. It has been proposed that the transport of GCase into the ER is differentially affected by the different leaders and the 19-AA leader peptide cleavage takes place when it enters the ER. Oligosaccharide modulations also occur but they do not affect the intracellular stabilization or the catalytic activity of GCase; hence, their significance remains unclear [29, 30]. The transportation of GCase to the lysosome is independent of mannose-6-phosphate as suggested by the following lines of evidence. In the lysosomal disease state, the transportation of lysosomal enzymes into the lysosome could not be targeted *via* the mannose-6-phosphate-dependent pathway [31, 32]. Studies in different animal and cellular models have suggested that LIMP2, which is responsible for GCase transportation to the lysosome, is independent of mannose-6-phosphate [26].

An important function of GCase is to degrade glucocerebroside (also known as glucosylceramide) into glucose and ceramide. It also potentially cleaves β-glucosides such as glucosylsphingosine. GCase deficiency renders glucocerebroside accumulation within cells of the reticuloendothelial system [21].

### The occurrence of *GBA* gene mutations: from GD to PD

The correlation between *GBA* mutations and a higher risk for developing PD was initially observed in GD clinics about 14 years ago [33, 34], when GD patients and their relatives, who were supposed to be *GBA* mutation carriers, were found to have a higher incidence of PD than the general population [35]. This finding promoted investigation of patients diagnosed at the GD clinic, which revealed a PD incidence of about 25% [36]. A survey conducted at the GD clinic (Jerusalem) elucidated a similar finding to the earlier report [6, 37, 38]. In 2004, it was reported that the first-degree relatives of GD patients had a much higher incidence of PD relative to the normal population [6, 38]. This was when various Parkinsonism-associated GD populations were screened for this gene mutation to assess the vital role of *GBA* in the PD pathogenesis worldwide [38]. Animal models for the analysis of *GBA* mutations in GD had been established before the recognition of its role as a risk factor for PD and now new models have been designed especially for the evaluation of the correlation between *GBA* mutations and PD. In addition, there is a wide range of cell-based designs using e.g., the pluripotential induced stem cells (iPSCs) [39, 40]. Broad Gaucher Registry statistics suggest that the risks for developing PD in GD patients are 9%–12% and 5%–7% before the age of 80 years and 70 years, respectively, although the *GBA* mutations occur in 5%–10% of PD patients [41]. This made *GBA* mutations the most significant genetic risk factor for PD [42]. Macrophages of GD patients are associated with glycolipid stress and load and are thus regarded as “Gaucher cells”, which are characteristic of the disease pathology. The single heterozygous mutations were originally believed to be harmless, but after analysis of early cases of Parkinsonism in GD patients, the heterozygous mutations were considered to present a substantial risk of developing PD [6, 43, 44].

The associations of *GBA* mutations with dementia with Lewy bodies (DLB) and Parkinson’s disease dementia (PDD) have also been well established. *GBA1* mutations are a risk factor for the development of DLB, suggesting some correlations among *GBA1*, dementia and parkinsonism. Patients with DLB are 8 times more likely to be the GBA mutation-carriers than controls. Such a risk is greater than that reported for PD, and seems to correlate with disease severity, earlier age onset and progression of disease. *GBA* is involved in the DLB pathology, but the precise cause of this predisposition is not known. Recently, the association of DLB with the PD-linked *SCARB2* has highlighted the significance of the lysosomal pathway in DLB. Genetically, DLB seems to be heterogeneous, with common risk factors and relatively rare contribution from pathogenic causative mutations [45, 46]. There is no significant difference between DLB patients with and without a *GBA* mutation in the neuropathological data, though the *GBA -* mutation-carriers show a reduced activity of GCase and more prominent lipid profile alteration in the brain. *GBA* expression is reduced in PDD and DLB cases in the caudate nucleus and the temporal cortex [36, 47]. PD mild cognitive impairment (MCI) and PDD are two types of cognitive impairment and the most prevalent and disabling non-motor symptoms of PD. MCI is the early warning signal of late dementia in PD. Research evidence has shown that about 30% of newly diagnosed PD patients have MCI, > 40% of PD patients with normal cognitive function will develop MCI within 6 years, and about 80% of patients may develop PDD at the late phase of PD. Various studies have suggested that the *GBA* mutations are more likely to be associated with PD-MCI [47, 48].

### *GBA* mutations associated with PD: a clear link

About 495 mutations and rearrangements across the exons of *GBA* are known to be associated with GD, including splicing mutations, frameshift mutations, point mutations, deletions, insertions and null alleles, and most of them are “missense mutations”. The most common mutations are point mutations including N370S (seen exclusively in type 1 GD patients in the Europe, USA and Israel) and L444P (observed worldwide) [21, 35, 38]. There is a 10%–30% probability of developing PD among the heterozygous mutation carriers at the age of 80, a 20-fold rise as compared to the non-carriers [49–53]. The idiopathic PD is undifferentiated from GBA + PD, with the only distinctive characteristic being the earlier age of onset and a greater incidence of cognitive features [49, 54, 55]. Moreover, the variant E326K is a notable example that is not considered a GD-causing mutation but a risk factor for PD [54, 56]. This implies a precise, and perhaps a different process by which mutations make their carriers prone to PD. The enzymatic activity of GCase is differentially influenced by different *GBA* mutations, as many of the mutations lead to no residual activity whereas others result in decreased activity [39, 45].

In comparison to GD, a smaller number of *GBA* mutations has been observed in PD patients (~ 130 *GBA* mutations) [33, 34]. Only certain mutations that are most generally linked with PD have been screened in several studies. Hence, the PD-related mutants that are less persistent could go unidentified. Generally, c.1448 T > C (L444P) and C.1226A > G (N370S) are the two most persistent mutations amongst others. They even contribute to 70%–80% of *GBA* mutants linked with PD in certain populations [57]. In a population of Colombian PD patients, an increased frequency of the variant K198E was observed in comparison with controls, consistent with the recent finding in GD patients [33]. Severe *GBA* mutations such as c.115 + 1 G > A (IVS2 + 1), c.84dupG (84GG), c.1297 G > T (V394L), c.1263del + RecTL, c.1342 G > C (D409H), and c.1448 T > C (L444P), appear to be correlated with a higher risk of PD development compared to the mild mutations such as c.84dupG (84GGG) and N370S [58]. p.T369M is one of the *GBA* variants that do not cause GD in homozygous carriers and may modify the GCase activity. In some studies, the substitution of p.T369M was associated with PD, while in other studies it had similar or increased frequency in controls. It is of interest that the *GBA* p.T369M substitution was demonstrated to be correlated with declined GCase activity in PD patients and controls compared to that in non-carriers. A recent meta-analysis showed that the p.T369M allele was associated with an increased risk for PD [59, 60]. Besides, the severe mutations mentioned above are also linked with an earlier onset age, and also have greater incidence and elevated cognitive ability involvement [58–62]. A previous study showed that PD patients with extreme *GBA* mutations had significantly worse motor symptoms together with some non-motor symptoms such as insomnia and rapid-eye-movement sleep disturbances, compared to subjects with moderate mutations or idiopathic PD [63]. Interestingly, *GBA* is nothing more than a risk element for PD. The latter implies that the disease will not develop in every carrier. The implications of all these mutations remain to be clarified. Information from a wide array of human studies shows that around 9.1% of *GBA* carriers will develop PD. Several reports have indicated that the prevalence of PD in GD patients at age of 80 is about 30%, but more research is needed to validate this data [50]. Homozygous *GBA* variant-linked patients that are affected by GD have a greater risk of developing PD and generally have an earlier age of symptom onset [63]. It is important to note that even in the case of severe mutations, most GD subjects do not develop PD. The heterozygous and homozygous *GBA* variant-carriers exhibit a 5- and 10-times greater risk of having PD, respectively [50, 64, 65]. *GBA* mutations are present in ~ 2%–30% of PD patients [66] (Table 1).

**Table 1:** Frequency of GBA mutations in various populations of PD patients

***GBA*** variant	Population	No. of participants		Carrier frequency (%)		***P*** value	Most common variants	Reference
		Cases	Control	Cases	Control			
N370S, L444P, T369M, IVS6, IVS10, K303K, R262H, E326K, Rec*Nci*1	Mixed	1325	359	12.6%	5.3%	–	E326K, L444P, N370S, T369M	[55]
N370S, c.84dupG, R496H, L444P, V394L, IVS2 + 1, RecTL, D409H	Ashkenazi	420	333	17.9%	4.2%	≤0.0001	N370S	[57]
N370S, IVS2 + A > G, K198T, L444P, R329C, Rec*Nci*1, c.84dupG	Canadian	88	122	5.7%	0.8%	0.48%	Rec*Nci*1	[67]
L444P, N370S	Chinese population in Singapore	331	347	2.4%	0.0%	0.06	L444P	[68]
L444P, N370S	Italian	395	483	2.8%	0.2%	0.0018	L444P	[69]
L444P, N370S, c.84dupG, V394L, IVS2 + A > G, R496H	Ashkenazi	99	1543	31.3%	6.2%	≤0.0001	N370S	[70]
L444P, N370S	Russian	330	240	2.7%	0.4%	0.038	N370S	[71]
*GBA* exons	Japanese	534	544	9.4%	0.1%	≤0.0001	R120W, Rec*Nci*1	[72]
*GBA* exons	Korean	277	291	3.2%	0.0%	0.01	N188S, R257Q, P201H, L444P, S271G	[73]
*GBA* exons	Portugese	230	430	6.1%	0.7%	–	N370S, N396T	[74]
L444P, R353W, F213I, N370S	PD patients in China's mainland	402	413	2.7%	0.0%	0.0007	L444P	[75]
N370S, G377S, L444P	Brazilian	65	267	3.0%	0.0%	0.037	L444P	[76]
L444P, R120W, Rec*Nci*1	PD patients in Taiwan region	518	339	3.1%	1.2%	0.07	L444P, Rec*Nci*1	[77]
*GBA* exons	Greek	172	132	4.7%	0.8%	0.048	H255Q, L444P	[78]
N370S, 84GG, L444P, IVS2 + 1G > A, V394L, D409H, del55bp, R496H	Ashkenazi	250	–	12.8%	–	–	N370S	[79]
LRRK2 (G2019S), *GBA* exons	North African (Morocco, Algeria, Libya, Tunisia)	194	177	4.6%	0.5%	0.01	N370S, L444P, Rec*Nci*1	[57]

### Evidence for the potential mechanisms of *GBA* mutation involvement in PD

#### Cross-talks among GBA mutations, GCase, and α-synuclein, and their roles in PD

There has been a significant attempt over the past couple of years to reveal the pathogenic function of *GBA* mutations in PD. Accumulating evidence has suggested the autophagic and endolysosomal pathway failure in PD [57]. The autophagic and endolysosomal pathways are essential for α-synuclein depletion, while the aggregation of α-synuclein is one of the defining characteristics of PD that renders dopaminergic neuronal death [80, 81]. *GBA* mutations can structurally alter the GCase protein, leading to the loss of function or reduced enzymatic activity. Theoretically, these effects may arise in many forms through the following pathways:

i. Failure of GCase to escape the ER,
ii. Failure of GCase to bind to its trafficking transporter LIMP2,
iii. Degradation of unstable and misfolded GCase by the proteasome,
iv. Failure of GCase to leave the Golgi,
v. GCase inactivity due to the mutations at the active site, and
vi. Degradation of GCase by the proteasome [82].

**Fig. 1:**
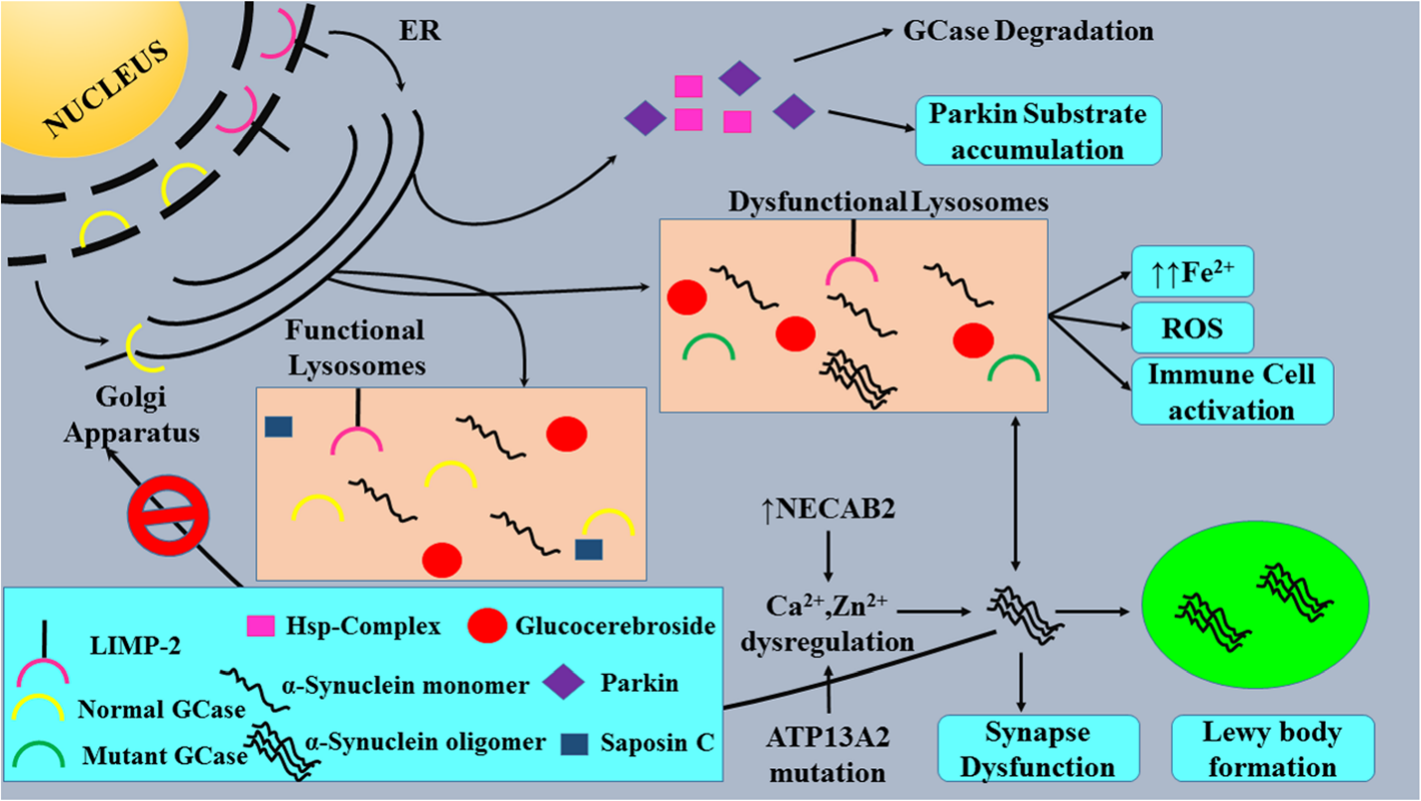
Feed-forward cascade of all the pathways that lead to PD development

All the pathways that lead to PD development are presented in Fig. 1.

Several recent studies have postulated a relationship between elevated levels of α-synuclein and diminished GCase activity in both *in vitro* and* in vivo* models. α-Synuclein deposition occurs in many forms in both the peripheral and central nervous systems. The progression of PD was proposed to be related to the expansion of α-synuclein aggregates between neurons. Neuronal cell lines overexpressing α-synuclein tend to release exosomes containing α-synuclein. Such exosomes will transfect regular neurons. The external α-synuclein may co-aggregate with native α-synuclein, ejected from the neuron, and transfect other neurons. Lysosomal impairment and deterioration of GCase expression facilitate the proliferation of α-synuclein aggregates [83, 84]. A correlated mutation study on all 72 vertebrate species with known complete sequences of α-synuclein and GCase revealed that α-synuclein and GCase probably co-evolve, and mutations could disrupt the beneficial interactions between them [85].

#### The association between GCase and α-synuclein

**Fig. 2:**
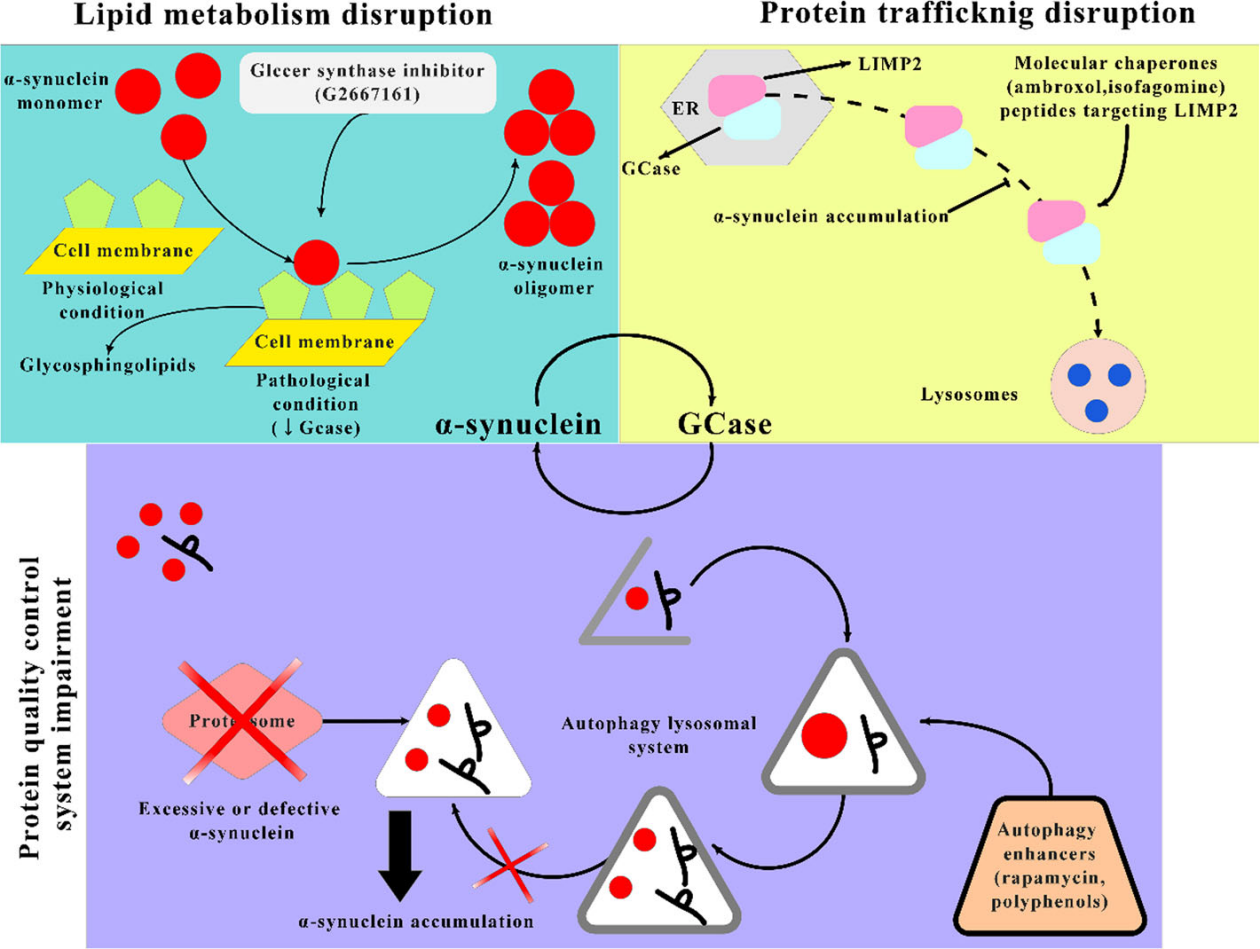
Mechanisms underlying the GCase–α-synuclein cross-talk that results in pathological conditions. GCase alterations induce disruptions of lipid composition and protein trafficking, inducing aggregation of α-synuclein

The relationship between α-synuclein and GCase particularly depends on the pH and the cellular site. The GCase substrate glucosylceramide could contribute to the aggregation of α-synuclein, which in turn results in decreased activity of GCase. *In vitro* experiments indicate that α-synuclein (membrane-bound) communicates with GCase in a different way from that between the unbound type of α-synuclein and GCase, creating a matrix that decreases the GCase activity at the lysosomal pH. The GCase–α-synuclein communication on the membrane surface involves a larger α-synuclein region than that in solution, and the bound α-synuclein (α-helix) acts as a mixed inhibitor for GCase. Generally, GCase binds to the lipid bilayer of the membrane where it is inserted partly. The active site of GCase possibly resides above the interface between the membrane and water. GCase is moved away from the membrane following contact with α-synuclein, which is membrane-bound. This can block access to substrates and disrupt the active GCase site. As a response, GCase shifts the attached α-synuclein helical residues farther from the bilayer and may have a detrimental effect on α-synuclein degradation by lysosomes. Saposin C is a protein co-factor that is usually required by GCase. It competes with α-synuclein for binding to the active site of GCase, thereby preventing some inhibition of GCase [86–89]. Transgenic mice expressing *GBA* L444P together with wild-type human *SNCA* have a 40% drop in GCase activity, which elicits the aggregation of α-synuclein in cortical neurons. The co-expression of *GBA* L444P and *SNCA* A53T leads to the exacerbation of the intensity of gastrointestinal and motor effects as compared to those expressing the *A53T* mutation alone [90]. Studies showing brain areas with aggregation of α-synuclein have implied a persuasive reduction in GCase function. This connection has been shown at the periphery, with correlations of lower GCase leukocyte activity with greater plasma levels of oligomeric α-synuclein. The mechanisms underlying the implication of the GCase–α-synuclein crosstalk in pathological conditions are presented in Fig. 2.

Under normal conditions, α-synuclein is unfolded and attains a tertiary arrangement on certain biochemical interactions. The abnormal aggregation of α-synuclein is toxic to the survival of dopaminergic neurons, contributing to the PD-associated neurodegeneration. *GBA* gene mutations and overexpression can affect the aggregation and conformational changes of α-synuclein. Moreover, some species can stimulate neuroinflammatory responses, which can spread the pathology of α-synuclein from one cell to another. Most importantly, there are growing data suggesting failure of the endolysosomal and autophagic pathways in PD [81]. These pathways are essential for α-synuclein degradation, while α-synuclein accumulation in the dopaminergic neurons is a key hallmark of PD. GCase plays an important role in the lysosomal degradation of α-synuclein and has close cross-talks with α-synuclein [80]. In patients with mutations that decrease GCase, the degradation of α-synuclein within the cell is impaired and lysosomal functioning is compromised, leading to increased levels of oligomeric α-synuclein, which results in dopaminergic neuronal death in PD. Such condition may be enhanced by ageing, which is accompanied by impaired functions of lysosome and increased concentrations of cellular α-synuclein. This is how mutant α-synuclein influences the survival of dopaminergic neurons, causing cell demise [5].

PD is associated with mutations in both *SNCA* (which encodes α-synuclein) and *GBA* [90]. Murphy *et al.* reported a decrease in the functions of brain GCase in the early phase of intermittent PD, which then remains unchanged during PD development. The production and function of GCase are decreased in brain areas with α-synuclein deposition. While the lysosome malfunction is important in these areas, it is not sufficient to induce neuronal death. It has been shown that the decreased activity of GCase in cultured neurons leads to the declined clearance and increased levels of α-synuclein. The declined GCase function in the lysosome is associated with glucosyl sphingosine and glucosylceramide substrate aggregation, the latter of which confers more cytotoxicity [80, 91].

#### Other potential pathways involved in GBA-associated PD

Many mechanisms have been proposed to be engaged in *GBA*-associated neurodegeneration. A recent study showed that treatment with a glucosylceramide synthase inhibitor miglustat to reduce glycosphingolipid accumulation and overexpression of *GBA1* to augment GBA1 activity can reverse the *GBA1* deficiency-induced destabilization of α-synuclein tetramers and related multimers and protect against the α-synuclein preformed fibril-induced toxicity in hDA neurons derived from GBA + PD iPSCs, which suggest therapeutic opportunities for GD and PD patients [91]. This study suggested a neurotoxic connection between GCase and α-synuclein that can partly describe the process underlying GBA + PD. Therefore, no research till date has demonstrated increased glucosylceramide production in the presence of heterozygote *GBA* mutation.

Another process through which *GBA* mutations lead to PD is the impairment of ER-associated degradation (ERAD) and cell death correlated with ER stress. The accumulation of α-synuclein will trigger ER tension, impede the ER-related substrate degradation, and restrict the ER-to-Golgi traffic [92]. Some PD-associated genes like *PARK-2* are involved in ERAD. Collectively, the ERAD and ER tension may significantly participate in the PD pathogenesis [93]. The consistent findings of ER in experiments with a few of the mutant types of GCase indicate that ER stress is often engaged in PD pathogenesis in some *GBA* mutation-carriers [94–96].

It has also been demonstrated that the aggregation of GCase in aggresomal-like structures is promoted *via* the interactions of mutated GCase with *PARK-2* [93]. The ER stress found in *GBA* mutation models may be attributed to the aggregation of α-synuclein, instead of aggregation of GCase. The disturbance of ceramide metabolism may also be implicated in the PD pathogenesis. Although Lewy bodies are the pathological signature of PD [97], they are also present in several other diseases. The genes governing such diseases have been noted (such as *SMPD1*, *GALC, PLA2G6, ASAH1*, and *PANK2*) to play a vital role in ceramide metabolism [98, 99].

Ceramide is linked to PD through its involvement in inflammation and stress-induced cell death [100, 101]. Hence the pathway of ceramide metabolism may be associated with the formation of LB in GBA + PD [98] (Table 2).

**Table 2:** Cell and animal models used in *GBA* mutation studies

Model	Model characteristics	Reference
Fibroblasts of patients	Carriers of heterozygous *GBA* mutations without or with PD	[102]
Fibroblasts of PD patients	Heterozygous *GBA* L444P and N370S mutations	[103]
Dopaminergic neurons derived from iPSC (patient midbrain)	Carriers of heterozygous *GBA* variant N370S in twins discordant for PD.	[104]
Mouse model	Heterozygous *GBA* variant L444P	[105]
SH-SY5Y cells (human α-synuclein)	siRNA knockdown of *GBA*	[106]
Mouse	*GBA* knockout	[107]
Mouse	*GBA* point mutations (D409H, N370S, D409V, and V394L)	[108]
Zebrafish	Deletion of 23 bp *GBA*	[109]
Mouse	*GBA L444P* mutation + α-synuclein A53T mutation	[90]
NSC and dopaminergic cells derived from the iPSC and fibroblasts of patients	Heterozygous *GBA* N370S mutations	[110]
Embryonic fibroblasts from mice	*GBA* heterozygous mutation	[111]

*ER* endoplasmic reticulum; *GCase* glucocerebrosidase; *LIMP2* lysosomal integral membrane protein-2

### Mitochondrial dysfunction, reactive oxygen species (ROS), and neuroinflammation in *GBA*-associated PD

Mitochondrial dysfunction, neuroinflammation, and excessive ROS formation are key processes that lead to dopaminergic neuronal death, wherein the mitochondrial dysfunction is involved in the pathophysiology of both familial and idiopathic PD [112, 113]. Evidence has suggested that α-synuclein interacts with the mitochondrial import proteins through a cryptic mitochondrial import signal [114]. Mutations of PTEN-induced putative kinase (*PINK1*) and *PARK2* contribute to the monogenic PD, and are assumed to affect mitochondrial function by elevating the susceptibility to toxins [115]. In a neuronopathic GD mouse model (K14-lnl/lnl), Ossellame et al. found that the proteasomal and autophagic pathways in astrocytes and neurons were impaired and that insoluble α-synuclein deposits occurred in neurons [107, 116]. Depletion in GCase function in cell studies led to a gradual deterioration of the potential of mitochondrial membrane needed for ATP output, fragmented mitochondria, respiratory complex function loss, and oxidative stress.

Ultimately, dysregulation of calcium occurred in the mitochondria, resulting in a distorted potential of the membrane [117]. In addition, ROS are generated after mitochondrial dysfunction, inducing persistent oxidative damage that may trigger α-synuclein misfolding and activate other deteriorating channels in the neuron [118, 119]. Accordingly, secondary mitochondrial dysfunction may arise from the primary lysosomal insult (i.e., GCase activity loss). Cellular disturbances including ROS, impairment of mitophagy, and ER stress can further deteriorate the cellular homeostasis and promote α-synuclein accumulation [120]. The transcript level of the antioxidant NQ01 has been found to be augmented in GD patients and *GBA* heterozygotes with or without PD, which may serve as a potential compensatory process. Thus, the GCase insufficiency can increase the oxidative stress of neurons. Elevating the levels of glutathione as an antioxidant may be a therapeutic strategy, which can be achieved by administration of *N*-acetylcysteine [121, 122].

Chemotactic factors are a central part of the process of neuroinflammation in that they can allocate immunologic facilitators to the inflammatory site [123, 124]. Iron is increased in PD patients in the substantia nigra. Excessive iron can normally be chelated by neuromelanin or ferritin. However, with natural ageing, the residual iron is stored in the brain. Excessive levels of iron induced by reactive nitrogen species or ROS can contribute to neuronal death [125]. Disruption of zinc homeostasis can aggravate or cause neurodegeneration* via* induction of protein misfolding and oxidative stress. Zinc augmentation also occurs in the substantia nigra of PD patients [126]. Mutations of *PARK9/ATP13A2*, which encode a zinc pump that brings zinc into the membraned components, are correlated with juvenile-onset PD. *ATP13A2* overexpression causes extracellular zinc tolerance and facilitates the exosomal transfer of α-synuclein [127, 128]. Silenced *ATP13A2* expression in primary neurons causes a decline in lysosomal chelation of Zn^2+^ and increases the expression of Zn^2+^ transporters. The increased Zn^2+^ leads to lysosome dysfunction that leads to the GCase–α-synuclein pathological cascade. Either chelation of zinc or improvement of *ATP13A2* expression can improve the phenotype [129, 130].

### Prospects for the treatment of *GBA*-associated PD

**Fig. 3:**
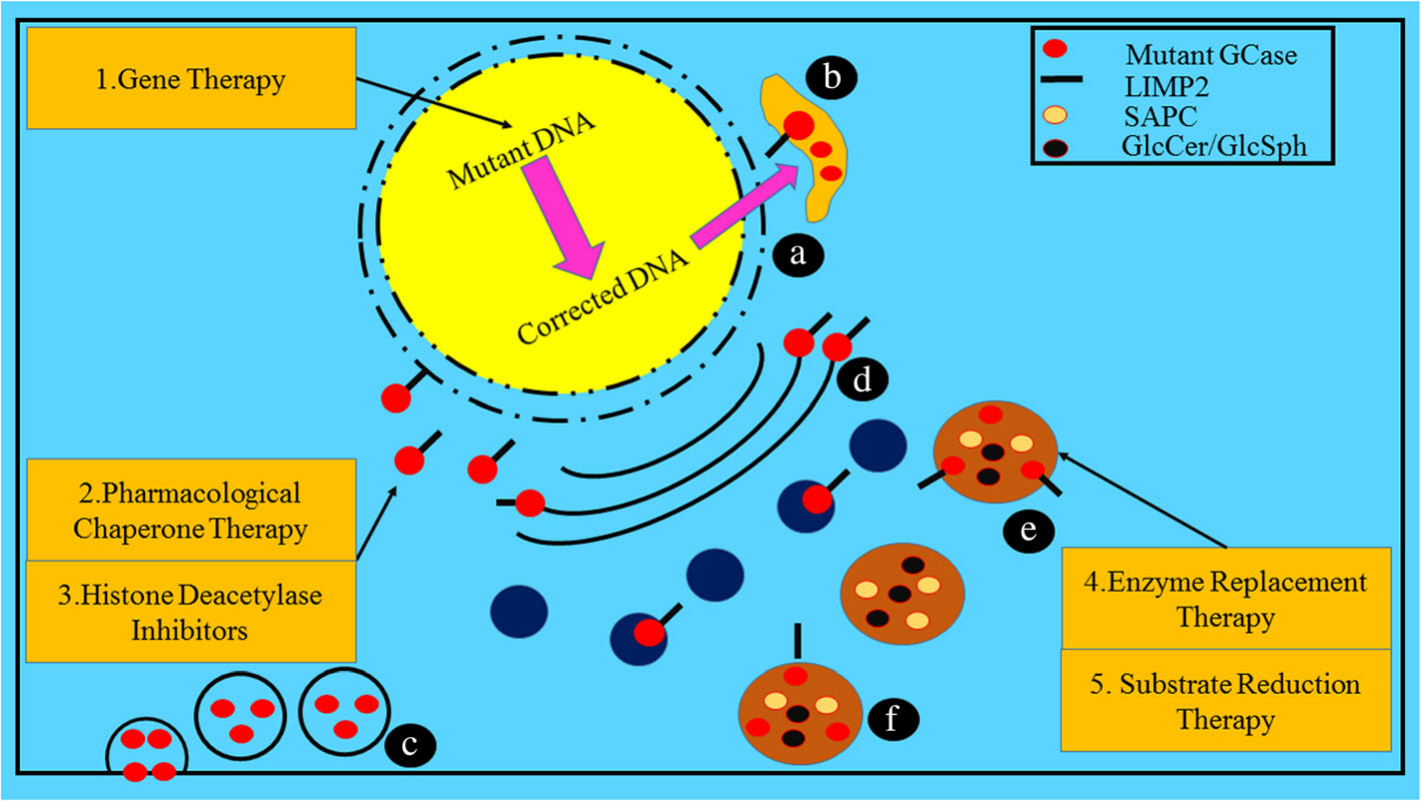
Distinctive hypothetical pathways through which impairment of GCase occurs and therapies targeting these pathways. **a**: GCase failure to escape the ER; **b** GCase failure to link with LIMP2 transporter; **c** misfolded and degraded GCase; **d** GCase failure to escape Golgi; **e** inactive GCase due to the mutation at the active site; **f** Altered GCase function due to the defective saposin C. The targeted therapies are (1) Gene therapy: direct replacement of mutant DNA with the correct DNA *via* viral infections; (2) Chaperone therapy to refold and stabilize misfolded proteins; (3) Histone deacetylase inhibitors that inhibit the response of unfolded protein; (4) Enzyme replacement therapy: substituting the dysfunctional enzyme with the recombinant enzyme in the targeted lysosome; and (5) Substrate replacement therapy: diminishing the accumulation of substrate independent of the enzyme level

Current promising therapies for GD include enzyme replacement therapy (ERT) and substrate replacement therapy (SRT), both have been approved by the FDA and work through the production and maintenance of a more standard ratio of GCase substrate in patients. These therapies have markedly enhanced the visceral symptoms of GD but are unable to gain access to the blood-brain barrier, so the neuronopathic symptoms of GD cannot be alleviated or reversed [2]. Considering the significant involvement of GCase in the PD pathogenesis, innovative therapies that can restore the levels of neural GCase, may not only enhance the life quality of neuronopathic patients, but also delay the development of PD in populations vulnerable to the GD-associated PD or idiopathic PD. Currently, brain-penetrating variants of SRT are under clinical trials with PD patients who are carriers of heterozygous *GBA* mutation [36, 129]. Numerous companies have been trying to tackle this problem, achieving very encouraging outcomes in animal and cell models. The interventions available in clinical trials so far mainly resolve pathways that are considered to be counterproductive in connecting *GBA* mutations to PD. The hypothetical pathways that lead to impairment of GCase and the associated therapies targeting these pathways are shown in Fig. 3.

One hypothesis demonstrates that the mutated GBA proteins are not adequately foldable in the ER, triggering protein accumulation in the cellular compartment, which induces dopaminergic neurons to respond to the stress, contributing to their damage and death. In addition, the β-GCase entanglement in the ER causes a declined enzyme level within the cell, inducing the aggregation of α-synuclein [102]. To target this pathogenic mechanism, distinctive chaperones, which are capable of facilitating the substrate replenishment, have been tested [103, 104, 131, 132]. A clinical trial on ambroxol, a chaperone that demonstrated extremely interesting preliminary findings, was established in 2016 (NCT02914366). This clinical trial at Phase 2 was aimed to evaluate the efficacy and safety of ambroxol to enhance the cognitive and motor characteristics of GBA + PD patients [125, 133]. Research has shown that administering chaperones to raise the native protein levels of chaperone could be the gateway to GCase refolding and restoration of normal enzymatic function in the brain. Arimoclomol is one of those chemical substances, which activates the heat shock reaction, thus magnifying Hsp70 and other proteins from heat shock. The administration of arimoclomol to fibroblasts from *L444P* genotype patients increased GCase activity at a frequency comparable to about 1 unit of the regular ERT drug, alglucerase [132]. Another pharmacological chaperone isofagomine, has been studied *in vivo* and *in vitro *to determine the capacity to modify the *GBA* mutation-induced phenotype and to stabilize the GCase [134].

Another pathway to be explored for the treatment of GBA + PD is the accumulation of glucosylceramide (substrate of GCase) in the dopaminergic neurons due to *GBA* mutation [131, 135, 136, 137]. Recently, a phase 2 multicenter, placebo-controlled, randomized, double-blind study was initiated to analyze the pharmacokinetics, pharmacodynamics, and safety of an oral molecule, ibiglustat (SAR402671), which is capable of reducing beta-GCase levels in early-stage GBA + PD. Compared with the wild-type form, mutated GCases are much more unstable. Modification of GCase degradation may be another effective technique to increase the enzymatic function and thereby combate aggregation of α-synuclein and neurodegeneration. Hsp90 is responsible for the breakdown of misfolded GCase, together with another heat shock protein PARK-2. In general, specific HSP inhibitors and histone deacetylase inhibitors (HDACis) can enhance the levels of GCase, limiting its degradation. HDACis, in effect, prevent contact between GCase and Hdp90 by hyperactivating one of its domains [36, 138, 139, 140]. It has been proposed that the inactivation of protein phosphatase 2A may reflect the possible process *via *which the deficiency of GCase blocks autophagy and facilitates the aggregation of α-synuclein. Autophagy upregulation by the mTOR inhibitors polyphenols and rapamycin showed favorable effects on synucleinopathy in animal and cell models by decreasing the intracytoplasmic aggregates of protein and ultimately the death of cells. Surprisingly, these pathways tend to underlie the cross-talk between α-synuclein and GCase and can influence the propagation of disease [136, 141, 142].

## Conclusion and future interventions

The most important genetic risk factor for PD was an unintended finding from GD patient trials. The identification of the link between *GBA* mutations and PD has given rise to vital implications and findings that advance the understanding of PD pathogenesis. The mechanism underlying the *GBA* mutation-induced elevated risk of PD has gained much attention in genetic research. A substantial body of evidence has supported the relationship between GCase and α-synuclein. GCase activity in PD patients is decreased in brain regions where α-synuclein builds up. The decreased activity of GCase causes neuronal aggregation of α-synuclein, which accelerates PD. Nevertheless, the lysosomal dysfunctioning has been widely investigated in PD following the initial findings. The endolysosomal pathway is implicated in α-synuclein accumulation and degeneration of dopaminergic neurons, in which a variety of genes observed in monogenic variants of PD or hereditary risk factors for the disease are involved, such as *ATP13A2, SNCA, PINK-1, PARK*, and *LRRK2*. GCase forms a complex together with membrane-bound α-synuclein, which decreases its activity. The α-synuclein–GCase complex also has a deleterious impact on α-synuclein degradation in the lysosome. There is an inverse association between α-synuclein levels and GCase activity and designed therapies that could increase the GCase activity may be useful for the treatment of PD. In addition, carriers of extreme mutations have faster disease advancement than mild mutation-carriers and homozygous mutations confer faster advancement than heterozygous mutations. The GBA + PD patients have an earlier onset age and are more likely to demonstrate cognitive dysfunction than PD patients without GBA mutation. The current promising therapies for GD are ERT and SRT, which have been approved by the FDA and are developed for the production and maintenance of GCase. These therapies have markedly enhanced the visceral symptoms of GD but have poor accessibility to the blood-brain barrier. Therapies directly targeting GBA + PD are now under clinical studies, such as chaperons (ambroxol, arimoclomol, and isofagomine), autophagy enhancers (rapamycin and polyphenols), ibiglustat (which reduces GCase levels), and GZ667161 (a glucosylceramide synthase).

## Data Availability

Not applicable.

## Abbreviations

ER: Endoplasmic reticulum
ERAD: Endoplasmic reticulum associated degradation
ERT: Enzyme replacement therapy
GBA: Glycosylceramidase beta
GBA+PD: GBA-associated Parkinson’s disease
GBAP: GBA pseudogene
GCase: Glucocerebrocidase
GD: Gaucher’s disease
HDACis: HSP direct inhibitors and histone deacetylase inhibitors
IPSC: Induced pluripotential stem cells
LB: Lewy body
LIMP2: Lysosomal integral membrane protein-2
MCI: Mild cognitive impairment
PD: Parkinson’s disease
ROS: Reactive oxygen species
SRT: Substrate replacement therapy
